# Intraoperative computerised tomography scan for percutaneous fixation of the pelvis: a retrospective case series

**DOI:** 10.1007/s00264-024-06265-7

**Published:** 2024-08-15

**Authors:** Monahan Kevin, Hogan William, Matthew Chilton, Maher Michael, Hughes Alice, Altman Gregory, Altman Daniel, Hammarstedt Jon Erik

**Affiliations:** Allegheny General Hospital, 320 E North Ave, Pittsburgh, PA 15201 USA

**Keywords:** Pelvis, CT scan, Intra-operative, O-arm, Fluoroscopy, Revision

## Abstract

**Purpose:**

Fractures and dislocations of the pelvic ring are complex injuries that when treating require meticulous attention to detail and often specialized technical skill. These injuries can be the result of high-energy trauma, particularly in younger patients, or low energy trauma more often found in the elderly. Regardless of mechanism, these injuries lie on a spectrum of severity and can be treated conservatively or surgically. Percutaneous fixation under fluoroscopic guidance is the preferred standard technique when treating these fractures. This technique can be challenging for a variety of reasons including patient characteristics, intra-operative image quality, fracture morphology, among others.

**Methods:**

This retrospective study evaluated the use of intra-operative computed tomography (CT) using an O-arm imaging system for critical evaluation of fluoroscopic-guided screw placement in twenty-three patients. We retrospectively reviewed all cases of patients who were treated by three fellowship-trained orthopaedic traumatologists during a one-year span. Patients undergoing percutaneous pelvis fixation using both standard fluoroscopy and intraoperative CT with the Medtronic O-arm® (Minneapolis, MN) imaging system. Additionally, procedures performed included open reduction internal fixation (ORIF) of the pelvic ring, acetabulum, and associated extremity fractures.

**Results:**

Twenty-three patients were included in this study. On average, the use of intraoperative CT added 24.4 min in operative time. Five patients (21.7%) required implant adjustment after O-arm spin. Fourteen patients underwent additional post-operative CT. No secondary revision surgeries were attempted after any post-operative CT.

**Conclusions:**

Our study suggests that intra-operative CT scan, compared to post-operative CT scan, can be utilized to prevent take-back surgery for misplaced implants and allow for adjustment in real-time.

## Introduction

Fractures and dislocations of the pelvis are challenging injuries for any orthopaedic specialist to treat. These patients may be involved in high energy traumas, resulting in multiple and possibly life-threatening injuries. This results in greater overall complexity in patient care; in addition, the bony anatomy of this region is complex and lies adjacent to viscera, large vasculature and neural elements which can make operative management technically challenging. Treatment with percutaneous reduction and fixation is preferential whenever possible, as open treatment with dissection and exposure may result in greater comorbidity.

Intraoperative fluoroscopy is the standard technique for guiding percutaneous fixation of pelvis fractures [1–4]. This technique requires the use of multiple fluoroscopic views and sequential imaging to guide and confirm reduction and implant placement during the procedure. This may be a demanding process for the surgeon, as well as the operating room and radiology staff [5, 6]. Other factors such as bowel gas and patient habitus may make visualization of important landmarks nearly impossible which increases the difficulty of the procedure [7]. Minor variations in sequential views can lead to mal-reduction, misplacement of instrumentation, and violation of anatomic structures. These mistakes, when discovered post operatively, can result in take backs to the operating room for revision or re-reduction. Therefore, improvements in technique with advanced imaging is a current area of inquiry [8–11]. In this case series, we present the utility of intraoperative CT in the form of an O-arm (without navigation) in the percutaneous placement of pelvis fixation as a supplemental imaging modality to check the accuracy of the reduction and implant placement prior to leaving the operating room. We predict this would not only eliminate the need for a return to the operating room but may also negate the need for a post-operative CT scan.

## Materials and methods

We retrospectively reviewed all cases of patients who were treated by three fellowship-trained orthopaedic traumatologists during a one-year span. Cases included patients undergoing percutaneous pelvis fixation using both standard fluoroscopy and intraoperative CT with the Medtronic O-arm® (Minneapolis, MN) imaging system. Additionally, procedures performed included open reduction internal fixation (ORIF) of the pelvic ring, acetabulum, and associated extremity fractures. Extremity fractures were not included in intra-operative CT scanning for review of implant placement. Standard surgical technique for percutaneous pin placement was utilized. Initial guide pin placement was performed using fluoroscopy. Implant placement was confirmed using a combination of standard fluoroscopic views of the pelvis. Once satisfactory reduction was achieved and implants were placed, intraoperative CT was used to confirm that no implants were in poor position and fractures were appropriately reduced. After intra-operative CT scan, cases that required adjustments underwent revision at that time to appropriately re-position implants and achieve successful osseous reduction. A post-operative CT scan was not routinely obtained in every case included in this study, and no post-operative CT scan led to revision surgery.

**Fig. 1:**
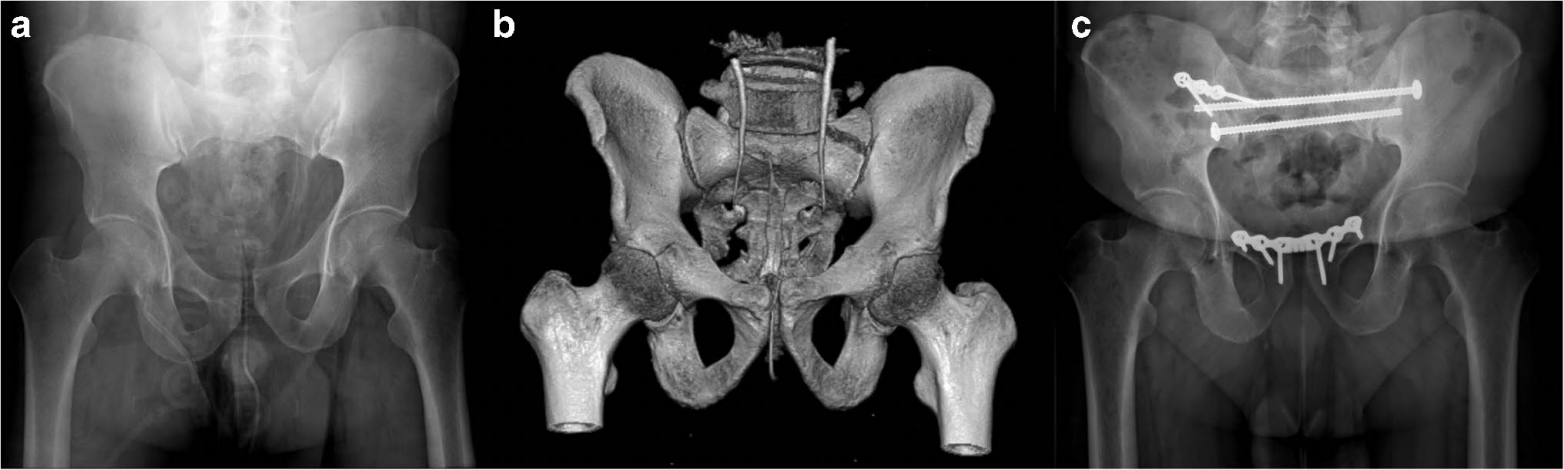
**A** Pre-operative anteroposterior (AP) pelvis and **B** 3-dimensional reconstruction of vertical shear injury the patient sustained falling approximately 40 feet from a water tower. **C** Final AP pelvis demonstrating open reduction internal fixation with combined sacroiliac fixation. Intra-operative O-arm scan demonstrated anatomic alignment of fractures with no evidence of mal-reduction or mal-positioned screw and plate placement

Data was collected via electronic and paper medical records including operating room flow sheets, operative reports, and saved imaging. Data collected included patient demographic information, date of surgery, injury complex treated, surgical procedure(s), total operating room time, time dedication to O-arm use, change in care due to O-arm, presence of dysmorphism, and comparison with standard pelvis CT. Those patients who underwent additional post-operative CT were used to compare radiation exposure to each patient as well as control to compare accuracy of intra-operative CT. To analyze radiation exposure, seven cases were found to have available data to compare intra-operative versus standard CT scanning of the pelvis using documented dose-length product (DLP) (Fig. 1).

## Results

Twenty-three patients were included in this study. Patient age ranged from 21–89 years. Fourteen (61%) patients were male and nine (39%) were female. Nine cases involved injuries isolated to the posterior ring, such as unstable sacral fractures or those fractures extending into the sacroiliac (SI) joint. Seven cases involved injuries in both the anterior and posterior ring of the pelvis requiring surgical intervention, such as severe anterior–posterior compression (APC) type injuries. Seven fractures involved injuries primarily to the posterior ring with an associated pubic rami fracture that required only posterior fixation (Table 1). Four cases also involved injuries requiring intervention at the same time, including fractures of the acetabulum, tibia, clavicle, and forearm.

**Table 1:** Characteristics of patients regarding individual injury pattern and treatment

Patient	Age/Sex	Fracture Pattern (Pelvic Ring, Sacrum)	Mechanism	Surgery	Total Time (min)	Change in care	Sacral dysmorphism
A	62 M	APC III- L APC II w/. R LC I	MCC	ORIF/CRPP	275	yes	No
B	54 M	L APC II, L 54 B-2	MCC	ORIF/CRPP	228	no	No
C	56 F	R 54B-2	MVA	ORIF/CRPP	198	no	No
D	32 F	R 54 B-2	Lawnmower accident	CRPP	211	yes	Yes
E	86 F	L LC-1	2-story fall	CRPP	58	no	No
F	48 M	R LC II	MCC	CRPP	157	no	No
G	26 M	R APC II, L anterior column acetabulum	MVA	ORIF/CRPP	252	no	No
H	51 F	L 54B-3	MVA	ORIF/CRPP	108	no	No
I	61 F	R LC-1	MVA	CRPP	78	no	No
J	52 M	L 54B-2	MVA	CRPP	143	no	No
K	52 F	54C-3	MVA	ORIF/CRPP	190	no	No
L	21 M	L 54B-3	Industrial equipment	CRPP	107	no	Yes
M	33 M	R LC-1, R 54B-3	MVA	CRPP	79	no	Yes
N	36 M	L Vertical Shear, 54C-2	Fall from water tower	CRPP	344	no	No
O	66 F	R LC I, R 54B-2	MVA	ORIF/CRPP	122	no	Yes
P	36 M	BL 54B-2	MVA	CRPP	194	yes	Yes
Q	26 F	R LCI	Fall from tree stand	CRPP	79	no	No
R	47 M	R LCI, R 54B-2	Fall from ladder	ORIF/CRPP	40	no	No
S	26 M	R LC I, R 54B-2	ATV	CRPP	258	no	No
T	63 M	L Vertical Shear, L 54B-2	Fall from roof	CRPP	165	yes	No
U	89 F	L LC1	GLF	CRPP/ORIF	85	no	Yes
V	23 M	R 54B-3	MVA	CRPP	73	no	No
W	22 M	L APC I, L 54B-2	MCC	CRPP	135	yes	Yes

*M* male, *F* female, *R* right, *L* left, *APC* anterior posterior compression *LC* lateral compression, *MCC* motorcycle collision, *MVA* motor vehicle accident, *ATV* all-terrain vehicle, *GLF* ground level fall, *ORIF* open reduction internal fixation, *CRPP* closed reduction percutaneous pinning, min (minutes)

The most common mechanism of injury was a motor vehicle accident (MVA) which accounted for 44% of patient injuries. Falls from various heights and motorcycle accidents were also found.

**Fig. 2:**
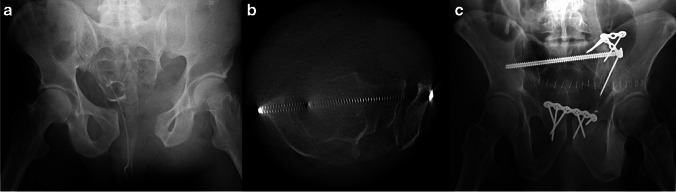
**A** Pre-operative AP radiograph demonstrating APC III pelvis injury to a patient after a motorcycle accident. **B** intra-operative O-arm scan demonstrated mal-reduction to the left sacroiliac joint as well as an incarcerated bone fragment. **C** Post-operative pelvic inlet radiograph demonstrating final fixation of pelvic ring injury

Five patients (21.7%) required implant modification after review of intraoperative CT (Table 2). The first case involved an intraoperative CT finding of an incarcerated fragment within the sacroiliac joint, resulting in transition to open reduction (Fig. 2). In two cases, the S1 iliosacral screw was prominent and shorted to an acceptable length. In the fourth case a longer screw was needed to fully compress the fracture site after the initial O-arm demonstrated incomplete fracture reduction. In the fifth case, the washer and screw head were incompletely compressed and re-adjustment of the screw and washer were necessary to ensure complete flushing of the washer against the sacrum to avoid significant hardware loosening and prominence.

**Table 2:** Complications related to initial fixation. Five patients required adjustment of fixation based on intra-operative O-arm scanning. All five patients did not require revision surgery after these initial corrections were made

Patient	Complication	Revision
A	Fracture fragment incarceration in SI joint	Required conversion to open reduction and removal of incarcerated fragment
D	Excessive Length/ Anterior Trajectory of SI screw	Shorter screw exchange with posterior trajectory adjustment
P	Excessive Length/ Anterior Trajectory of SI screw	Shorter screw exchange with posterior trajectory adjustment
T	Short Screw length/ Incomplete fracture compression	Longer partially- threaded screw placed
W	Washer incompletely compressed w/ prominent head of screw	Washer/screw compressed to bone

The presence of sacral dysmorphism was evident in seven patients (30.4%) within this series and in 3/5 of the above cases requiring implant modification (Tables 1 and 2). The amount of additional time required for use of O-arm was only documented five times. On average, the use of intraoperative CT added 24.4 min to total operative time.

Fourteen patients underwent additional post-operative CT. No secondary revision surgeries were attempted after any post-operative CT. The surgeons in this case series used post-operative CT less frequently as time progressed and ultimately discontinued using it, as intra-operative CT with O-arm was deemed sufficient in later cases. In a limited number of cases, the average dosage for intraoperative CT and standard pelvis CT were 251.04 mGy-cm and 364.00 mGy-cm, respectively.

## Discussion

The utility of intraoperative CT for placement of percutaneous pelvic fixation was supported in this retrospective case series. In our study, five patients required revision after an O-arm scan demonstrated unsatisfying results in fracture alignment and/or implant placement. Each case was successfully revised under the same anesthetic event and, although not documented in all cases, O-arm use added approximately twenty-five minutes to each case. Of all patients in this study, 61% underwent a post-operative CT scan. No additional patients required revision surgery after post-operative CT scan suggesting the O-arm scan successfully identified complications preventing secondary take-back to the operating room.

Percutaneous fixation of pelvic ring injuries represents a minimally invasive, effective technique although technically difficult given the complex anatomy in this area. Intraoperative CT has not supplanted fluoroscopy. Traditional techniques using fluoroscopy are historically safe and effective and have been described and used in several series for screw placement [2, 5, 10–12]. However, percutaneous fixation of pelvic ring injuries is not without complications such as malreduction, screw malposition, etc. These complications have been documented anywhere from 0.5%-29.3% with associated neurologic deficits of 7.7% but ranging up to 23.5% in select studies [1, 8, 10, 13–18]. Routt et al. [12] demonstrated complications relating to patient body habitus, poor injury understanding, and surgical technique. A review article by Zwingmann et al. [8] demonstrated malposition rates of screws of 2.6% using conventional fluoroscopic techniques. In a review article from a German pelvic trauma database, complication rates with conventional fluoroscopic guidance were noted to be as high as 29.3% [14]. Given the wide variability in the aforementioned complication rates, improving the accuracy of percutaneous screws would serve to decrease and narrow these numbers.

In a study looking at efficacy of post-operative CT scan for implant evaluation, Elnahal et al. [17] demonstrated an overall 6% revision rate of implants after percutaneous pelvic ring fixation. When isolated to patients involving posterior SI fixation, 22% of patients were found to have mal-positioned metal with 4% requiring revision after postoperative CT scan. The conclusion of this study was that routine use of post-operative CT scan in patients with posterior SI joint fixation has value, especially in patients with suspicious findings on intraoperative radiographs. Additionally, postoperative CT scans have been shown to correlate well with mal-positioned implants when neurologic deficits are present [16–19]. Given that CT scans add significant value in evaluating implants and reduction, we advocate that, if available, intraoperative CT may provide more efficient care by negating the need for a post-operative CT and repeated visits to the operating room for implant modification. Ultimately, if prominent or misplaced hardware is detected, it is up to surgeon discretion for how to proceed. In cases of obvious malposition, we recommend intraoperative adjustment. However, in cases of borderline or uncertain positioning, the surgeon should determine the best course of action. A return to the operating room and a second anaesthesia event is unquestionably an undesirable patient outcome that we must look to improve upon.

Both of these prior studies [17, 18] advocate for post-operative CT scans to more completely evaluate suspicious placement of screws. While our study makes no direct comparison to intra-operative versus post-operative CT scan it does suggest that, if available, intra-operative CT scan may be more efficient. Post-operative CT scan requires significant logistical demands such as patient transport to adjacent CT suites suitable for trauma patients that may be in critical condition. Additionally, in this study seven patients underwent both intra-operative O-arm scan and post-operative CT and in all but one patient the radiation dose was lower from O-arm use. This suggests that O-arm use alone could prevent the increased radiation exposure from a routine post-operative CT scan [17, 20].

Those factors that make fluoroscopic visualization more challenging, such as habitus or bowel gas/contrast, would be an obvious indication for intraoperative CT use. Morbid obesity can make visualization of vital structures in the pelvis during fluoroscopic fixation difficult without expert fluoroscopy technicians and also can cause significant difficulty with standard operative instrumentation [8, 14]. Intra-abdominal contrast agents may also cause significant barriers to visualization [12]. Additionally, sacral dysmorphism can certainly make percutaneous fixation using conventional methods more difficult. While this study does not offer a solution to these fluoroscopic barriers, it does offer a more immediate confirmation of implant positioning. Intra-operative O-arm scanning may prevent unnecessary patient exposure to another round of anesthesia, reintubation as well as the cost and morbidity associated with a second, separate surgery. Additionally, the average effective dose of radiation was lower with intraoperative CT in our series, but the clinical implications of this difference is unclear.

Although the utility of intraoperative CT was supported in this series, our study does have significant limitations. It is a relatively small retrospective review. It is subject to biases of treating surgeons and limitations of documentation. This study did not address the use of navigated CT for treatment of pelvic ring injuries which may be more useful in patients with prohibitive body habitus, bowel gas or associated injuries that make fluoroscopic visualization nearly impossible. Instrumented navigation has shown early promising results and will likely continue to be the focus of future research as it becomes more widely available and used [21–26].

## Conclusion

In summary, intraoperative CT scan provided excellent results for fixation of pelvic ring injuries in this small case series. No patients required a return to the operating room for a second procedure. Although this article does not intend to propose the use of routine intraoperative CT scanning for complex fluoroscopic pelvis fixation, it does demonstrate the potential for successful use. Post-operative CT scanning comes with additional risks and costs that may possibly be prevented with CT scan at the time of surgery. Further research involving intraoperative O-arm scanning should be aimed at analyzing cost, radiation exposure, and overall surgical time effects when utilized during pelvic ring surgery. Additionally, the use of navigated fixation of pelvic ring injuries continues to evolve and may play a role in more complex cases due to patient body habitus, anatomy, and fracture classification.
